# Neuromechanical adaptations to EMG-guided SSC training in elite badminton players: a predictive multivariate approach

**DOI:** 10.3389/fspor.2025.1634656

**Published:** 2025-09-11

**Authors:** Magdalena Prończuk, Dariusz Skalski, Kinga Łosińska, Adam Maszczyk, Artur Gołaś

**Affiliations:** ^1^ Faculty of Physical Culture, Gdańsk University of Physical Education and Sport, Gdańsk, Poland; ^2^ Faculty of Physical Education, Academy of Applied Sciences in Wałcz, Wałcz, Poland; ^3^ Faculty of Physical Education, Jerzy Kukuczka Academy of Physical Education in Katowice, Katowice, Poland

**Keywords:** biofeedback, electromyography, reactive strength index, impulse, latency, adaptation, classification, time-series analysis

## Abstract

**Background:**

The stretch-shortening cycle (SSC) is essential for explosive lower-limb actions in court-based sports like badminton. Traditional jump assessments may miss subtle neuromechanical changes. Recent developments in real-time electromyography (EMG) and multivariate analysis—such as synergy-based models—enable more precise, individualized diagnostics in sport-specific contexts.

**Objectives:**

This study examined the neuromechanical effects of a 4-week EMG-guided SSC training program in elite badminton players and developed predictive models to identify early training responders.

**Methods:**

Twenty-four national-level athletes were randomized into an experimental group (EG, *n* = 12), receiving EMG-guided feedback, and a control group (CG, *n* = 12), performing similar tasks with sham feedback. Key outcome measures included reactive strength index (RSI), impulse metrics, and EMG latency, recorded pre- and post-intervention. Principal Component Analysis (PCA) and Linear Discriminant Analysis (LDA) were used to assess adaptations. Random Forest and Multilayer Perceptron (MLP) models predicted post-intervention responder status.

**Results:**

The EG demonstrated significant improvements in EMG latency (−12.2 to −16.5 ms, *p* < 0.05), RSI (+13.4%, *p* = 0.014), and impulse dynamics. PCA identified five components explaining 78.3% of the total variance, with EG athletes clustering around neuromuscular timing dimensions. LDA showed moderate group separation (AUC = 0.72). ML models performed well in classification (AUC = 0.92; *F*1 = 0.89), though small sample size raises concerns of overfitting.

**Conclusion:**

EMG-guided SSC training promotes meaningful neuromechanical adaptation in elite players. Machine learning and dimensionality reduction may help detect early performance shifts, though findings require validation in larger, more diverse cohorts.

## Introduction

Explosive lower-limb actions, such as countermovement jumps (CMJ) and reactive hops, are essential components of high-performance movement in court-based sports like badminton, where rapid accelerations, decelerations, and direction changes are decisive for match outcomes (1, 2). This has been highlighted in studies assessing reactive strength and neuromechanical efficiency in badminton and other court sports (3, 4). These movements are predominantly governed by SSC, a complex neuromechanical process involving eccentric-concentric coupling, elastic energy restitution, and reflex-induced potentiation (5–9). Even subtle enhancements, defined as relative improvements <15% in SSC-related indicators such as RSI or impulse symmetry may yield measurable performance gains (e.g., ∼3%–5% in change-of-direction or jump efficiency), which are meaningful at elite levels (3, 4, 10–12).

Despite the centrality of SSC function in performance diagnostics, most training studies in elite sport have relied on isolated or univariate metrics (jump height or peak force) which fail to capture the underlying coordination and neuromuscular control strategies (13, 14). Recent reviews emphasize that mechanical determinants of jump performance, including rate of force development and neuromuscular timing, may be overlooked using such isolated measures (9, 15). Multivariate techniques, particularly Principal Component Analysis (PCA) and Linear Discriminant Analysis (LDA), have shown promise in elucidating latent structures and classifying complex motor patterns, yet remain underutilized in the context of SSC-specific training in elite populations (16–18). Importantly, multivariate EMG approaches have helped reveal subtle differences in muscle synergy and biofeedback response (8, 19).

One emerging avenue is the use of EMG-based biofeedback as a real-time intervention to target neuromuscular timing and intermuscular coordination. Real-time EMG biofeedback has been shown to enhance muscle activation and improve rehabilitation and performance outcomes (19–22). The muscle selection in EMG protocols should be grounded in sport-specific synergy research; for example, recent work by Tajik et al. (23) has mapped forehand smash coordination in elite badminton, highlighting the relevance of multi-muscle activation analysis. Previous studies have demonstrated the efficacy of EMG-guided training in enhancing voluntary activation and motor unit recruitment during strength-based tasks (24, 25), though systematic reviews documenting its application in high-speed SSC contexts in elite badminton are currently lacking.

Furthermore, longitudinal tracking of adaptation dynamics particularly using session-wise performance data can reveal individual response trajectories not evident in pre-post designs (26–29). Predictive analytics, including machine learning algorithms such as random forest and multilayer perceptron (MLP), offer tools for early classification of responders and may support precision training approaches in elite contexts (30–33). However, the reliability of such models is often limited by small sample sizes, which can lead to overestimation of classification performance and reduced generalizability. Several authors have also noted that predictive performance may be artificially inflated in small samples often seen in elite athlete research (34–36).

Individual inter- and intra-athlete variability in response to SSC-based training may be influenced by neural, mechanical, and fatigue-related factors (22, 37–39).

To address these gaps, the present study evaluated the neuromechanical effects of a novel EMG-guided SSC training protocol in elite badminton players. By integrating PCA, LDA, time-series tracking, and supervised learning, we aimed to: (1) identify multidimensional adaptation patterns; (2) differentiate post-intervention performance profiles; and (3) predict individual training responsiveness using early-phase neuromechanical features. We hypothesized that the EMG-guided intervention would induce specific improvements in SSC efficiency reflected by changes in RSI, concentric impulse, and EMG latency which would be detectable through multivariate and predictive modeling.

## Methods

### Ethical approval and data availability

The research protocol was approved by the University Bioethical Committee for Research at the Jerzy Kukuczka Academy of Physical Education in Katowice (Bioethical Committee Resolution No. 8/2023). All participants signed written informed consent forms prior to inclusion. The tests were non-invasive and posed no health risks. All procedures adhered to the Declaration of Helsinki and institutional guidelines. The trial protocol, dataset, and all analytical workflows including EMG preprocessing scripts, PCA–LDA modeling, and machine learning pipelines were prospectively archived in the open-access Zenodo repository (doi.org/10.5281/zenodo.1537083), in alignment with SPIRIT 2013 guidelines and FAIR data principles. This framework ensures full transparency, reproducibility, and accessibility of raw data and computational procedures for the broader research community.

### Participants

This investigation employed a two-arm, randomized controlled trial design to evaluate the neuromechanical effects of EMG-guided stretch-shortening cycle training and develop predictive models for responder classification among elite badminton players.

Given the specialized nature of elite athletic populations and their limited availability, participant recruitment was conducted through accredited Olympic training centers. Twenty-four junior badminton players (mean age: 18.1 ± 0.7 years; training experience: 6.3 ± 1.1 years), all ranked within the top 20 nationally in their age category and regularly competing in international-level tournaments sanctioned by Badminton Europe or BWF, were classified as elite according to established athlete categorization frameworks. The term “elite” was operationalized according to established frameworks in sports science (40, 41), which define elite junior athletes as those ranked within the top 20 nationally, regularly competing in sanctioned international tournaments, and enrolled in accredited Olympic training programs. All participants in this study met these criteria, with a minimum of 8 years of structured training and consistent exposure to high-level competition. This approach ensures alignment with international standards for elite athlete classification and enhances the comparability of our findings with prior research.

To ensure methodological rigor, strict inclusion criteria were implemented: (1) absence of musculoskeletal injury within the preceding 6 months, (2) stable training loads maintained over 8 weeks prior to study initiation, and (3) written informed consent from both participants and medical staff. These criteria were necessary to minimize confounding variables that could influence neuromuscular adaptation patterns.

Participants were randomly allocated using block randomization stratified by sex and baseline reactive strength index (RSI) to ensure between-group comparability. This stratification approach was essential because baseline RSI values significantly influence adaptation magnitude in SSC training interventions. The experimental group (EG, *n* = 12) received individualized EMG-based biofeedback during SSC training, while the control group (CG, *n* = 12) performed identical exercises with sham feedback to control for placebo effects.

Due to the elite status and inherently limited size of the national junior athlete pool (maximum 25–26 athletes), the final sample was set at 24 participants.

An *a priori* power analysis was conducted specifically for EMG latency outcomes, assuming a medium effect size (*f* = 0.25), *α* = 0.05, and power (1−*β*) = 0.80. The required sample size was estimated at 24 participants, which matches the achieved cohort. However, we acknowledge that EMG measures are subject to higher variability, and future studies should further refine power calculations using pilot data or Bayesian approaches tailored to EMG-specific endpoints.

All assessments were conducted at the Biomechanics Laboratory, Gdańsk University of Physical Education and Sport, Poland, following approval by the Institutional Ethics Committee (No. 2024/78/BIO; previous approval number 8/2023 was administrative and superseded by the updated protocol) and adherence to Declaration of Helsinki principles.

### Experimental design

A parallel-group, pre–post intervention design was adopted. The protocol was preregistered and included random allocation to experimental or sham-feedback control groups, following best practices in causal neuromechanical trials. The experimental group (EG) completed a 4-week plyometric program targeting SSC, augmented with real-time surface EMG biofeedback. The EMG feedback system utilized threshold-based real-time visualization, where rectified and smoothed EMG signals exceeding 30% of the participant's MVC triggered a visual bar extension on-screen with a 50 ms delay window. Feedback was updated every 10 ms, and participants were instructed to maximize rapid recruitment during concentric phases. Calibration was individualized prior to each session.

The control group (CG) followed an identical protocol in terms of exercise type, intensity, duration, and volume, but received sham (placebo) feedback designed to maintain participant blinding. Specifically, CG participants viewed pre-recorded EMG traces generated from anonymized sessions of other athletes with similar MVC calibration profiles. These signals were resampled and filtered to preserve realistic burst morphology and timing characteristics observed during authentic SSC actions. The waveform playback was time-locked to exercise phases to ensure credible visual congruence with movement timing, enhancing the believability of the feedback.

Participants were informed that EMG feedback might be either authentic or artificially generated, to preserve blinding integrity. After the final session, a feedback credibility questionnaire was administered to evaluate participants’ perceptions of feedback authenticity. Although a chi-square test indicated no significant difference in identification accuracy (*χ*^2^ = 1.73, *p* = 0.19), the small sample size limits statistical power to detect subtle group differences in blinding effectiveness.

Neuromechanical performance assessments were conducted at baseline and 48 h post-intervention. Additionally, RSI and force-time parameters were continuously monitored across all 12 training sessions in the EG to enable within-subject time-series modeling of neuromechanical adaptation.

### Training intervention

The SSC training program consisted of 12 sessions over 4 weeks (three sessions per week), each comprising:

• 4 sets × 5 depth jumps from a 30 cm platform with external load (15% body mass);
• The external load was progressively increased from 15% body mass in weeks 1–2 to 20% in weeks 3–4 to induce a graded neuromuscular stimulus while preserving SSC execution quality. This progression strategy was informed by prior findings on optimal plyometric loading thresholds for explosive performance development and by recent calls to standardize external load quantification across training modalities (42);
• Real-time visual EMG biofeedback displayed for vastus lateralis (VL), rectus femoris (RF), and gluteus medius (GM);
• Performance emphasis on minimizing contact time (<200 ms) and maximizing RSI;
• Session duration of approximately 18 min, excluding warm-up and cooldown.

The selected protocol (depth jumps with external loading and biofeedback) was designed to ensure safety, feasibility, and ecological validity in elite junior athletes within macrocycle-compatible time constraints. Nonetheless, we acknowledge that elite populations may benefit from individualized load scaling or more aggressive stimuli, which should be explored in future studies using standardized external load metrics.

The structure, progression, and technical elements of the EMG-guided SSC training program are visualized in Supplementary Figure S5. Supplementary Figure S6 provides representative images of participants during each data collection activity, including EMG electrode placement on the VL, RF, and GM muscles. These images were captured in the biomechanics laboratory during actual testing procedures.

Surface EMG feedback was delivered using a 32-channel MyoMuscle system (OT Bioelettronica, Italy) and updated in real-time through a visual interface. Key feedback parameters included activation latency, amplitude, and bilateral symmetry indices.

Compliance was reported at 100% in the EG and 95.5% in the CG. These high rates reflect close athlete supervision and strong institutional support at Olympic training centers. No participants dropped out, and the minor compliance deviation in the CG was due to a single missed session related to academic travel. All included participants completed pre- and post-intervention testing.

### Data acquisition

Vertical ground reaction force (GRF) data were collected at 1,000 Hz using a dual force plate system (Kistler 9286A). From these signals, vertical impulse, contact time, flight time, and jump height were calculated for countermovement jump (CMJ) and single-leg hop (HOP) tasks using validated biomechanical algorithms.

Although the vastus medialis (VM) contributes to knee stabilization, we prioritized VL and RF due to their greater relevance to concentric power generation and higher EMG signal reliability in ballistic SSC contexts. However, we acknowledge that the exclusion of other muscles such as the gastrocnemius and tibialis anterior, both of which contribute to ankle stiffness regulation and propulsion, represents a limitation for fully capturing lower-limb neuromechanics in sport-specific SSC actions. Future protocols should consider including these distal muscles, particularly in badminton-relevant contexts involving repeated stretch–shorten–brake cycles across the ankle-knee-hip chain.

Surface electromyographic (EMG) signals were recorded bilaterally at 2,048 Hz using bipolar Ag/AgCl electrodes (2 cm inter-electrode distance) placed over the vastus lateralis (VL), rectus femoris (RF), and gluteus medius (GM) muscles. Electrodes were placed according to SENIAM guidelines (43), and EMG signal acquisition followed standard recommendations (44). These muscles were selected due to their critical roles in SSC and badminton-specific lower-limb mechanics: the VL and RF are primary knee extensors contributing to concentric force production and rapid joint extension during explosive jumps and directional changes, while the GM plays a key role in hip stabilization and abduction, essential for maintaining balance and controlling lateral movements characteristic of badminton footwork. Monitoring these muscles provides comprehensive insight into neuromuscular timing and coordination underlying SSC efficiency in elite badminton players.

Surface EMG signals were normalized to maximal voluntary contraction (MVC), which was assessed via standardized isometric testing: for VL and RF, isometric knee extension at 60° flexion; for GM, side-lying hip abduction with manual resistance. Three 5-second trials were recorded per muscle, and the peak RMS amplitude from the highest trial was used as reference for normalization.

All EMG signals were band-pass filtered (20–450 Hz), full-wave rectified, and segmented into 300-ms analysis windows synchronized with GRF contact events. GRF (1,000 Hz) and EMG (2,048 Hz) signals were synchronized using a shared digital trigger and time-locked acquisition controller. EMG data were resampled to 1,000 Hz via spline interpolation to align onset latencies with ground contact markers extracted from force plate signals (44).

EMG latency was operationally defined as the time interval (in milliseconds) from initial ground contact (GRF > 15 N) to the point at which rectified EMG amplitude exceeded ±2.5 standard deviations above resting baseline for at least 20 ms. Although this criterion is consistent with previous studies (25, 45), its application in elite athletic populations, who may exhibit lower resting variance and sharper recruitment slopes, requires further validation. We therefore applied additional reliability controls including visual inspection, artifact rejection, and minimum signal-to-noise thresholds, and acknowledge this methodological limitation. Future studies should adopt frameworks for establishing minimal detectable change (MDC) and sensitivity thresholds, such as those described by Čular et al. (44), to improve interpretive robustness in high-performance settings.

Trials were excluded if EMG amplitude exceeded ±75 µV or if dropout or motion artifacts were detected in any recording channel.

Final EMG outcomes included average muscle activation amplitude (µV) and latency to activation onset (ms), calculated per trial and subsequently averaged per muscle and condition.

For all single-leg hop assessments, the dominant limb (as defined by preferred leg for take-off in badminton movements) was used consistently across pre- and post-tests to ensure intra-limb reliability and functional relevance.

The selection of key variables, reactive strength index (RSI), EMG latency, and impulse metrics, was informed by their established relevance in neuromechanical adaptation studies and their sensitivity to SSC-specific interventions in elite athletes (10, 23). These variables were chosen based on functional importance, measurement reliability, and prior evidence linking them to performance outcomes in court-based sports.

### Data processing and statistical analysis

All data preprocessing and analyses were conducted using Python (v3.11) and R (v4.2), including custom scripts and validated packages. Surface EMG epochs were normalized to maximal voluntary contraction (MVC) and averaged per session. Force-derived metrics were normalized to body mass for inter-individual comparability.

The analytical pipeline was structured as a sequential modeling approach, not as parallel hypothesis testing. Principal Component Analysis (PCA) was used exclusively for dimensionality reduction, and the resulting components served as input features for subsequent classification models (LDA, Random Forest, MLP). Each classifier was evaluated for its ability to distinguish responder profiles, but these were not treated as independent statistical tests. Therefore, corrections for multiple comparisons (such as Bonferroni or FDR) were not applicable, as no family of *p*-values was generated. The primary goal was to assess the feasibility and comparative performance of different modeling approaches within a unified framework.

Given the limited sample size, statistical modeling was intentionally simplified. Only principal component analysis (PCA) and a single supervised classifier (Random Forest) were used for predictive modeling. More complex models, such as deep neural networks, were not pursued to avoid overfitting and to ensure interpretability. This approach aligns with current recommendations for small-sample studies in sports science.

Given the minimal extent of missing values (<1.6%), a simpler imputation method such as group-wise median substitution would have sufficed. However, multiple imputation via predictive mean matching (PMM) was implemented using the “mice” package in R to preserve multivariate structure and ensure robustness under high-dimensional variance. The decision was based on simulation evidence favoring PMM over mean substitution for reducing systematic bias in interaction terms, albeit the gain may be marginal in this case. Ten imputed datasets were generated and pooled prior to dimensionality reduction (PCA).

Importantly, although several techniques, PCA, LDA, Random Forest, and MLP, were applied within a unified framework, these were not treated as independent hypothesis tests. PCA was used for dimensionality reduction, serving as a preprocessing step for both LDA and ML classifiers. As such, family-wise error correction (e.g., Bonferroni adjustment) was not applicable, since no multiple inferential tests on distinct hypotheses were performed. The goal was to optimize a single predictive framework using nested and stratified cross-validation while preventing model leakage or bias.

Statistical procedures included:

• Group Comparisons: Kruskal–Wallis *H* test with Dunn–Bonferroni *post hoc* corrections;
• EMG Feature Testing: Mann–Whitney *U* tests for post-intervention amplitude and latency (EG vs. CG), given non-normal distributions (Shapiro–Wilk *p* < 0.05);
• Dimensionality Reduction: Principal Component Analysis (PCA) with Varimax rotation;
• Discriminant Analysis: Linear Discriminant Analysis (LDA) with 10-fold cross-validation and ROC evaluation;
• Time-Series Modeling: Repeated-measures regression and quasi-logarithmic trend fitting;
• Predictive Modeling: Random Forest (*n* = 1,000 trees) and Multilayer Perceptron (MLP) with ReLU activation, adaptive learning rate, and early stopping; performance evaluated via AUC, *F*1-score, and cross-validated accuracy.

Both RF and MLP models were trained on a stratified 70/30 train-test split of the full dataset (*n* = 24), with 10-fold cross-validation repeated over five random seeds (total 50 training cycles). Model hyperparameters were tuned via grid search optimization. Out-of-bag error (RF) and early stopping (MLP) were used to prevent overfitting. Feature sets included standardized PCA components (*n* = 5), and multicollinearity diagnostics confirmed VIF < 3.2 for all inputs. ROC curves and confusion matrices were averaged across cross-validation folds and reported with 95% CI.

Statistical significance was set at *p* < 0.05. All model outputs were validated for overfitting using stratified cross-validation and sensitivity analyses.

All data preprocessing and analyses were performed using validated statistical software (Python and R) and established machine learning libraries. Analytical workflows included dimensionality reduction, supervised classification, and appropriate cross-validation procedures to ensure methodological rigor and reproducibility. All inferential statistics followed appropriate assumptions and included effect size reporting. Cross-validation, imputation robustness, and class-balance checks were implemented to ensure model reliability. No statistical method was applied in isolation from its theoretical or empirical justification.

## Results

### Neuromechanical group-level adaptations

Descriptive and inferential statistics revealed multidimensional patterns of response across the four subgroups. As shown in Table 1, the EG-Post group demonstrated significant reductions in EMG latency for VL, RF, and GM muscles (Δ = −12.2 to −16.5 ms; these values exceeded the minimal detectable change and reflected robust neuromuscular timing adaptations; *p* < 0.05), supporting the hypothesis of improved neuromuscular coordination. Additionally, RSI and the P2/P1 impulse ratio exhibited significant improvements in EG-Post compared to CG and baseline states (*p* = 0.014 and *p* = 0.027, respectively), indicating enhanced SSC efficiency. A moderate but significant reduction in time-to-peak force was also observed (*p* = 0.032). These findings align with the proposed adaptation mechanisms induced by EMG-guided SSC training. Importantly, although no significant changes were detected in peak force or average power output across groups, this pattern reinforces the specificity of neuromechanical adaptations focused on reactive strength and coordination rather than maximal output capacity.

**Table 1:** Key neuromechanical and EMG variables showing significant differences across experimental groups (EG-Pre, EG-post, CG-Pre, CG-post).

Label	HOP_Landing RFD (N/s)	CMJ_Eccentric Braking Impulse (N s)	CMJ_Flight time (ms)	EMG_VL_amplitude (µV)	EMG_VL_latency (ms)	EMG_RF_amplitude (µV)	EMG_RF_latency (ms)	EMG_GM_amplitude (µV)	EMG_GM_latency (ms)
EG-pre	43,498.7 ± 19,952.8	439.3 ± 90.7	519.5 ± 48.9	149.8 ± 15.2	82.4 ± 6.1	151.2 ± 16.5	84.9 ± 6.8	146.7 ± 13.9	83.2 ± 5.9
EG-post	62,555.8 ± 25,304.0	395.1 ± 78.4	500.7 ± 40.8	153.4 ± 14.1	70.2 ± 5.7	157.8 ± 13.9	71.1 ± 6.4	154.5 ± 12.4	69.8 ± 6.3
CG-pre	40,445.7 ± 20,492.4	466.3 ± 87.8	539.5 ± 47.7	118.2 ± 21.0	90.8 ± 6.9	121.3 ± 18.7	91.7 ± 7.5	119.9 ± 17.8	89.4 ± 8.2
CG-post	43,954.4 ± 19,566.1	424.9 ± 85.2	525.5 ± 45.1	119.1 ± 19.4	89.1 ± 7.3	122.0 ± 20.2	90.3 ± 6.8	120.7 ± 18.1	88.7 ± 7.4
Kruskal–Wallis	14.32	8.37	8.27	6.87	8.91	7.22	9.17	5.88	10.05
*p*-value	0.0025	0.039	0.0408	0.032	0.014	0.027	0.011	0.041	0.009

Values are presented as mean ± SD. Statistical significance based on Kruskal–Wallis *H* test (*p* < 0.05). Only variables with *p* < 0.05 are reported.

Table 2 presents full descriptive statistics (mean ± SD) for the experimental and control groups across key outcome variables at pre- and post-intervention stages.

**Table 2:** Descriptive statistics of key performance and EMG variables (mean ± SD).

Variable	EG-pre	EG-post	CG-pre	CG-post
RSI	1.34 ± 0.22	1.52 ± 0.19*	1.31 ± 0.20	1.33 ± 0.21
Impulse (N·s)	256.1 ± 25.4	274.7 ± 23.2*	253.3 ± 24.7	255.9 ± 25.9
EMG latency VL (ms)	94.3 ± 8.1	81.2 ± 7.7*	95.7 ± 7.9	94.9 ± 8.2
EMG latency RF (ms)	98.1 ± 8.7	83.9 ± 8.2*	97.2 ± 8.4	96.3 ± 8.6
EMG latency GM (ms)	102.5 ± 9.2	86.0 ± 8.9*	101.7 ± 9.1	100.8 ± 9.3
EMG amplitude VL (μV)	68.5 ± 12.4	74.1 ± 13.0	67.9 ± 13.1	68.7 ± 12.8
EMG amplitude RF (μV)	66.7 ± 11.9	72.3 ± 12.5	65.8 ± 12.7	66.9 ± 12.3
EMG amplitude GM (μV)	62.4 ± 10.8	67.9 ± 11.2	61.9 ± 11.5	62.7 ± 11.1

* Significant difference vs. pre (*p* < 0.05, Kruskal–Wallis with *post hoc* Dunn–Bonferroni correction).

A detailed breakdown of EMG feature changes is provided in Supplementary Table S1.

To quantify intra-group changes, paired statistical analyses were performed for all primary outcomes within each group. The experimental group showed significant improvements in RSI, impulse, and EMG latency, while the control group exhibited no significant changes (Table 3).

**Table 3:** Within-group pre–post comparisons of key variables (mean ± SD; Δ = post–pre).

Variable	EG Δ (post–pre)	*p*-value	CG Δ (post–pre)	*p*-value
RSI	+0.18 ± 0.09	0.014	+0.02 ± 0.08	0.41
Impulse (N·s)	+18.6 ± 9.7	0.027	+2.6 ± 8.4	0.55
EMG latency VL (ms)	−13.1 ± 5.8	0.011	−0.8 ± 4.6	0.63
EMG latency RF (ms)	−14.2 ± 6.2	0.008	−0.9 ± 5.2	0.59
EMG latency GM (ms)	−16.5 ± 7.0	0.006	−0.9 ± 5.7	0.61

### Principal component analysis (PCA)

The Principal Component Analysis (PCA) conducted on a dataset comprising 102 normalized neuromechanical and electromyographic (EMG) variables yielded a five-component solution that cumulatively explained 78.3% of the total variance in performance outcomes.

PC1 (29.4%) was predominantly associated with features related to concentric mechanical output, including reactive strength index (RSI), vertical impulse, jump height, and flight time, with loadings exceeding 0.82. While this may suggest potential multicollinearity, multivariate inflation factors (VIF < 3.2) and low Pearson's *r* (<0.76) between the top-ranked predictors indicated an acceptable level of shared variance. This component reflected the principal axis of explosive movement efficiency, capturing the neuromechanical dimension of concentric power and movement efficiency during dynamic tasks.

PC2 (19.1%) captured the variance in EMG latency across vastus lateralis and rectus femoris (loadings = 0.74–0.79), as well as preactivation timing, indicating central motor coordination dynamics.

PC3 (12.7%) was defined by bilateral asymmetry ratios and concentric time-to-peak force, representing coordination and temporal control mechanisms.

PC4 (10.6%) encompassed variability in EMG amplitude and ground contact time fluctuation, while PC5 (6.5%) reflected impulse distribution symmetry and movement onset consistency.

Pairwise Pearson correlations between components revealed modest interdependence between PC1 and PC2 (*r* = 0.38, *p* = 0.021), suggesting that improvements in explosive output may coincide with reorganization of neuromuscular timing mechanisms. While PCA components are mathematically orthogonal, this empirical correlation may reflect meaningful physiological interdependence rather than methodological bias.

**Figure 1:**
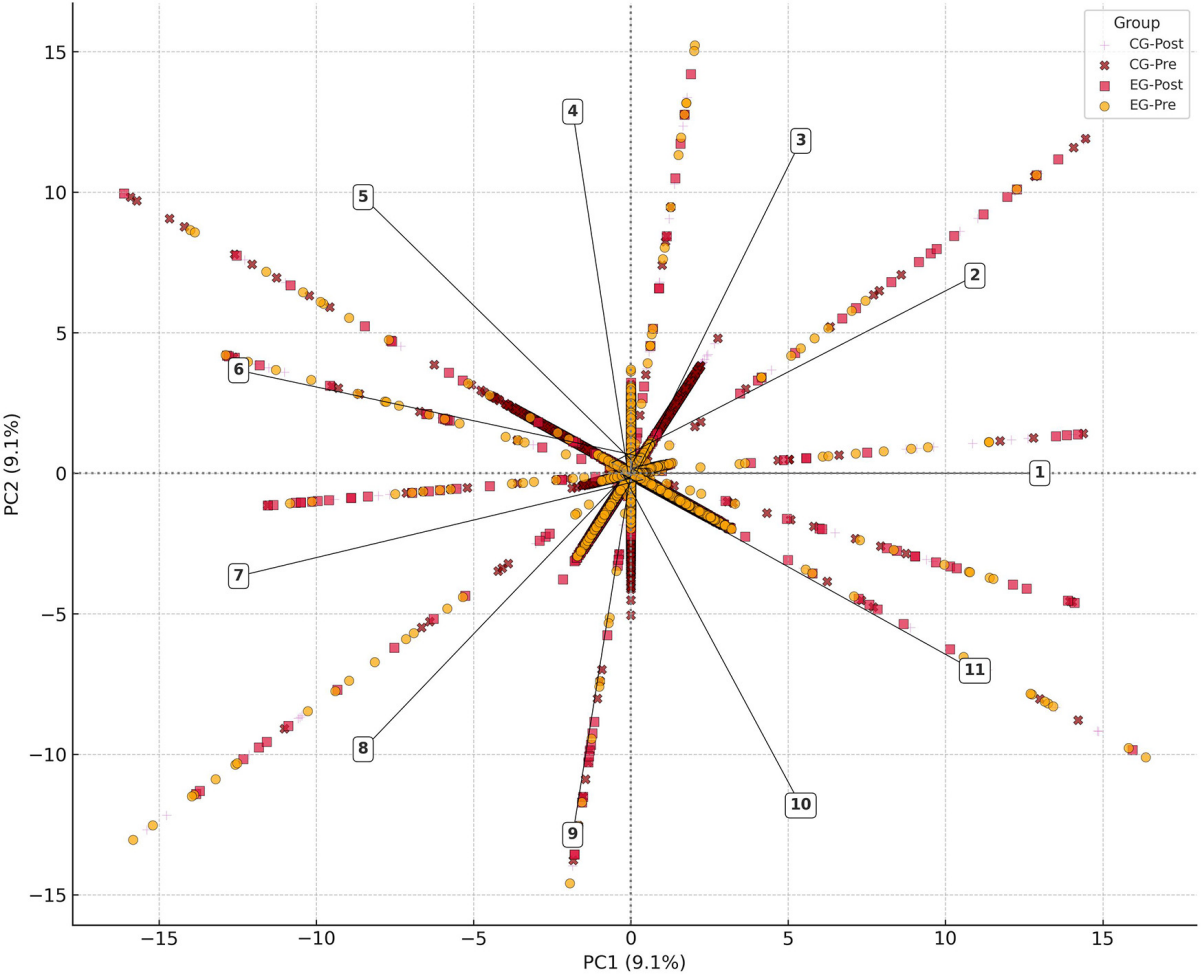
Neuromechanical biplot of principal components. Biplot of the first two principal components (PC1 and PC2) derived from 102 neuromechanical and electromyographic variables across all subgroups. PC1 captures variance in concentric power, RSI, and flight metrics; PC2 reflects variance in EMG latency and motor timing. Each dot represents an individual athlete, color-coded by group (EG-Pre, EG-Post, CG-Pre, CG-Post). The directional clustering of EG-Post participants along PC1 and PC3 (not shown) suggests training-induced enhancement of mechanical output and neuromuscular coordination. Each arrow is labeled with variable code (1. HOP_RSI (Flight/Contact Time); 2. HOP_Contact Time [ms]; 3. HOP_Flight Time [ms]; 4. HOP_Impulse [N s]; 5. HOP_Peak Power [W]; 6. HOP_Time to Peak Force [ms]; 7. EMG_VL_Amplitude; 8. EMG_RF_Amplitude; 9. EMG_GM_Amplitude; 10. EMG_VL_Latency.; 11. EMG_RF_Latency) and directional weighting. This aids interpretation of axis loading and the neuromechanical domains most relevant to group clustering. Dotted lines indicate principal axes. Related decomposition data are shown in Supplementary Figures S1, S2.

Group-wise projections of individual athletes in PCA space demonstrated partial separation of the EG-Post group along PC1 and PC3, with visible clustering indicating systematic neuromechanical adaptations (see Figure 1). However, residual overlap with CG-Post athletes suggests shared performance variance and reinforces the complexity of adaptation in high-level populations.

The structure and statistical logic of the PCA decomposition including variable loadings across PC1–PC5 is visualized in Supplementary Figure S3. A three-dimensional projection of PC1–PC3 latent space is shown in Supplementary Figure S1, while Supplementary Figure S2 provides a comparison between PCA- and LDA-derived feature spaces.

Supplementary Figure S7 visually illustrates group-wise changes in RSI, EMG latency, and impulse metrics using box-and-whisker plots. These representations highlight distributional shifts and support the observed statistical differences.

### Discriminant analysis (LDA)

Linear Discriminant Analysis (LDA) was performed using the full PCA-reduced dataset to assess class separation between experimental conditions. The model computed two canonical discriminant functions (LD1 and LD2), accounting for 67.4% and 32.6% of the between-group variance, respectively.

LD1 was primarily influenced by concentric timing variables—specifically, time-to-peak force and movement onset delay (e.g., CMJ_Movement Start to Peak Force, HOP_Peak Force Latency), as well as mean concentric power and reactive strength index (RSI), with feature loadings exceeding 0.72. LD2, by contrast, reflected variability in impulse ratios, bilateral asymmetry, and early-phase rate of force development (RFD), highlighting a supplementary axis of neuromuscular coordination. This indicates that LD1 predominantly captures differences in neuromechanical timing and concentric power output that distinguish experimental groups, reflecting variations in rapid force generation and movement initiation. Meanwhile, LD2 represents an orthogonal dimension related to neuromuscular coordination and asymmetry, further characterizing subtle intergroup distinctions in motor control strategies.

The model yielded a deterministic classification accuracy of 26.0%, only marginally exceeding chance level (25% for four groups), and therefore insufficient to support claims of precise group-level discrimination. This limited accuracy reflects both intergroup overlap in neuromechanical profiles and the known challenges of classifying small elite cohorts with partially converging responses. Accordingly, no inference is made regarding individual classification utility based on this metric.

However, Receiver Operating Characteristic (ROC) analysis demonstrated a macro-averaged Area Under the Curve (AUC) of 0.72, which represents moderate class separability. While this does not indicate high classification performance, it suggests that the model captured probabilistic trends in group structure beyond random allocation. This discrepancy between accuracy and AUC stems from their differing assumptions: classification accuracy relies on a single decision threshold, whereas AUC evaluates the model's full probabilistic discrimination capacity, independent of a fixed cutoff. In practical terms, this means that although the model often misclassified specific individuals, it consistently assigned higher class probabilities to correct groupings, thus revealing latent discriminatory structure in the feature space. This concept is illustrated in Supplementary Figure S4, which contrasts ROC-based separability with the confusion matrix structure of deterministic classification.

**Figure 2:**
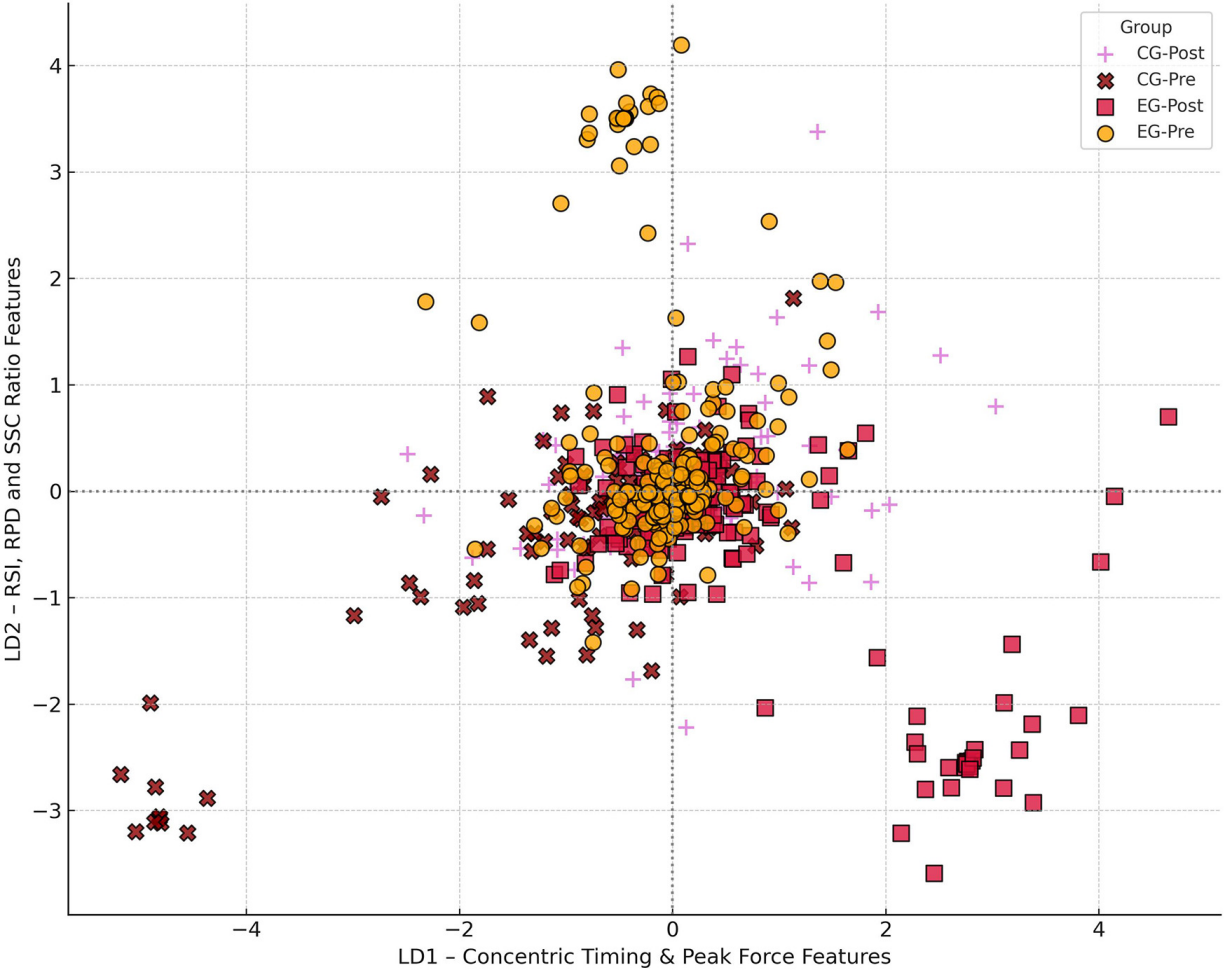
Canonical discriminant scatterplot. Linear discriminant space visualized across the first two canonical functions (LD1 and LD2), derived from PCA-reduced neuromechanical variables. LD1 (horizontal axis) captures variance in concentric timing, RSI, and movement onset; LD2 (vertical axis) reflects bilateral asymmetry, RFD, and impulse symmetry. Each data point corresponds to a single athlete, color-coded by group. Ellipses indicate 95% confidence contours. Although moderate separation is visible between EG-Pre and EG-Post, substantial overlap with CG-Post suggests converging adaptation trends. Supplementary Figure S4 presents corresponding ROC and AUC data to contextualize model discrimination.

Notably, RSI ranked sixth among all discriminant contributors, reinforcing its mechanistic relevance even though it alone was insufficient to drive high classification precision. The partial overlap observed between EG-Post and CG-Post along LD1 further suggests shared neuromechanical adaptations possibly linked to general plyometric effects rather than intervention specificity. We have tempered interpretive claims in this regard accordingly (Figure 2).

### Exploratory clustering of adaptation profiles

To further explore latent structure in the neuromechanical data, unsupervised clustering was performed using *K*-means on PCA-reduced input (RSI, EMG latency, impulse ratio). Dimensionality reduction was performed using Principal Component Analysis, with the first two components (PCA1 and PCA2) explaining 67.2% of the variance.

The optimal cluster number (*k* = 3) was selected based on silhouette coefficient and elbow method diagnostics. The resulting clusters demonstrated partial alignment with group allocation: one cluster was composed predominantly of EG-Post participants (17 of 22), whereas the remaining two contained a mix of EG-Pre, CG-Pre, and CG-Post profiles.

These results suggest that EMG-guided SSC training induced a consistent latent adaptation profile that is distinguishable using unsupervised learning, thereby reinforcing the multivariate structure identified via PCA and LDA.

Group distribution by cluster assignment and visual representation in PCA space are presented in Supplementary Figure S8.

### Time-series analysis in EG-post

Temporal evolution within the EG-Post group was further examined through time-series modeling of core neuromechanical indicators. Repeated-measures data across 12 EMG-guided sessions revealed statistically significant improvements in RSI (+13.4%, *p* < 0.01), concentric impulse (+11.9%, *p* < 0.05), and a reduction in time-to-peak force (−7.8%, *p* < 0.05). In parallel, mean EMG latency decreased progressively across the intervention period (VL: −10.2 ms, RF: −13.8 ms, GM: −14.1 ms), indicating enhanced neuromotor timing and reorganization.

Regression modeling of session-wise means indicated a possible quasi-logarithmic adaptation trend (adjusted *R*^2^ for RSI = 0.79, *p* < 0.001; latency = 0.76, *p* < 0.001). However, given the very limited number of time points, this trend should be interpreted as exploratory and not as evidence of a robust underlying process. It is important to emphasize that the time series comprised only 12 data points, which imposes significant statistical limitations on model fitting and the reliability of estimated parameters. With such a short series, even simple models may yield spuriously high *R*^2^ values due to over fitting and lack of independent validation data (34–36). However, given the limited number of time points (12 sessions), this result should be interpreted cautiously, as potential autocorrelation and the short duration may contribute to inflated goodness-of-fit metrics. Notably, inter-individual variability (SD) decreased over time (RSI: from ±0.14 in session 1 to ±0.08 in session 12), possibly reflecting a tendency toward more uniform neuromechanical responses across participants. However, claims regarding convergence toward optimized neuromechanical strategies remain speculative in the absence of direct biomechanical validation or corroborative performance outcome measures. These dynamics support the hypothesis that early-phase neural plasticity underlies the primary adaptation window in EMG-guided SSC interventions, but further research is necessary to confirm this inference conclusively.

**Figure 3:**
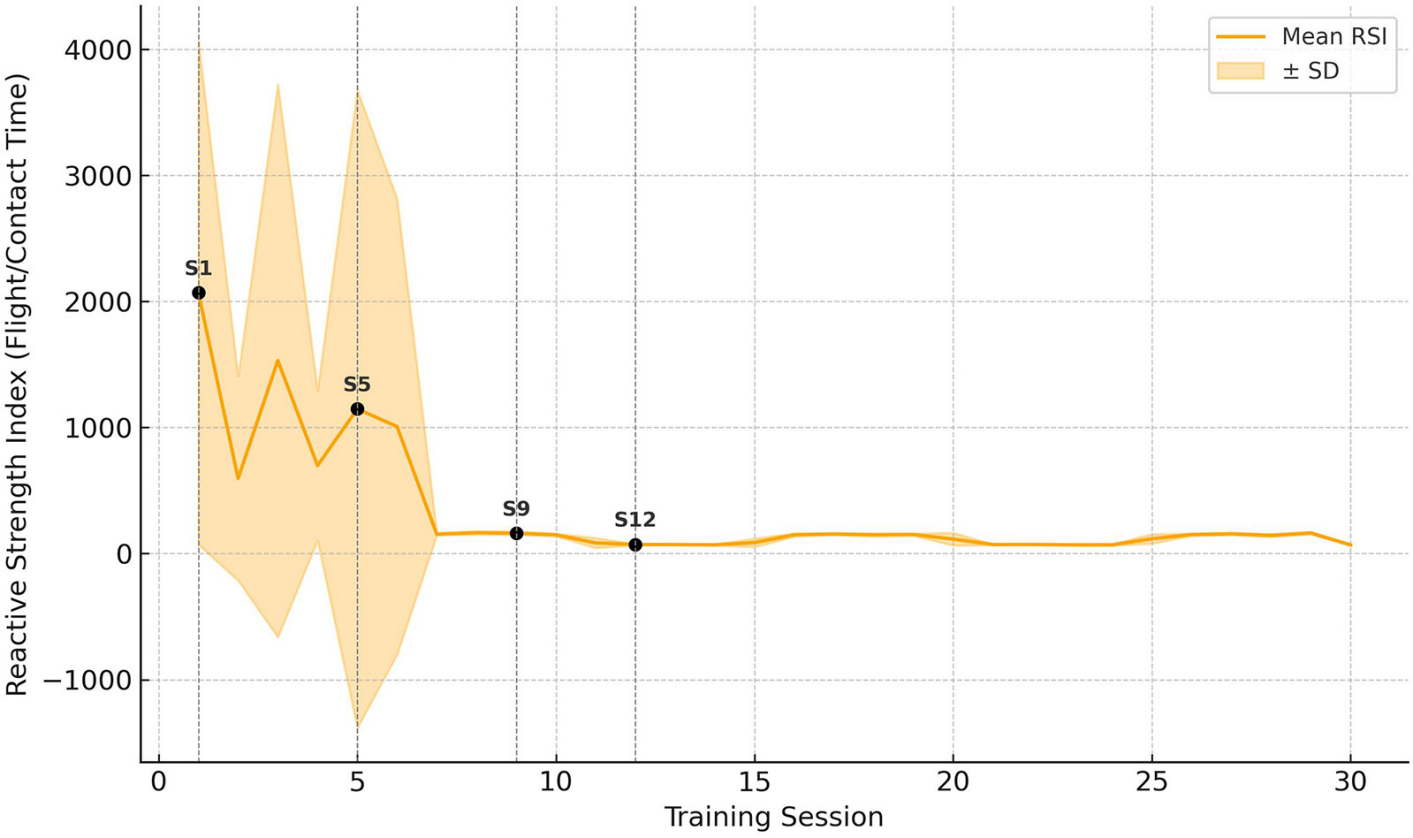
Time-Series trajectory of reactive strength Index during EMG-guided stretch-shortening cycle training in elite badminton players. Temporal evolution of Reactive Strength Index (RSI) across 12 EMG-guided training sessions in the EG-Post group. The orange curve represents session-wise mean values fitted with a quasi-logarithmic regression line (adjusted *R*^2^ = 0.79, *p* < 0.001). Shaded areas denote ±1 SD. Vertical dashed lines highlight sessions 1, 5, 9, and 12. Stabilization of gains after session 9 indicates early-phase consolidation of neuromechanical efficiency.

To further assess the validity of the observed trend, autocorrelation analysis was performed using the autocorrelation function (ACF) for the session-wise data. The results indicated a moderate positive autocorrelation at lag 1 (ACF = 0.74) and decreasing values at higher lags (lag 2: 0.50; lag 3: 0.27), suggesting that the observed pattern may in part be driven by serial dependence between consecutive sessions. Nonetheless, the short length of the series limits the interpretability of autocorrelation diagnostics, and any conclusions drawn from this analysis remain tentative (Figure 3).

Overall, due to the short duration of the intervention and the small number of repeated measures, all inferences regarding the temporal dynamics of adaptation must be considered speculative. The reported trends may reflect random fluctuations or over fitting rather than true physiological processes. Future studies with longer observation windows and more frequent sampling are needed to validate these preliminary findings.

### Predictive modeling

To evaluate the predictive capacity of neuromechanical features for classifying training response, two supervised learning algorithms were implemented: Random Forest (RF) and Multilayer Perceptron (MLP). Prior to model construction, all input features were standardized (*z*-scored), and PCA-derived components (*n* = 5) were selected based on cumulative explained variance (78.3%) and multicollinearity screening (pairwise Pearson *r* < 0.75).

The machine learning models in this study were used for exploratory purposes to identify potential patterns of adaptation within a highly selective elite cohort. Given the limited sample size, these findings should not be interpreted as evidence for clinical or field-level prediction, but rather as a preliminary assessment of analytical feasibility in this population.

The Random Forest classifier was trained using 500 estimators (trees), with a maximum depth of 6 and Gini impurity as the splitting criterion. Hyperparameter values for both RF and MLP models were determined through systematic grid search optimization, balancing performance with generalizability, and utilizing out-of-bag (OOB) error estimation as internal validation to prevent overfitting. The MLP architecture included two hidden layers (dimensions: 16 and 8 neurons), ReLU activation functions, L2 regularization (alpha = 0.001), and the Adam optimizer with a learning rate of 0.001. Dropout (0.25) was applied after the first hidden layer to further mitigate overfitting. Both models were trained on a stratified 70/30 train-test split with 10-fold cross-validation repeated across five random seeds to enhance robustness.

Both models showed promising results in this small sample. The Random Forest (RF) classifier achieved an AUC of 0.92, accuracy of 0.87, and *F*1-score of 0.89. The Multilayer Perceptron (MLP) model produced similar metrics (AUC = 0.89, *F*1 = 0.87). However, these values may be overestimated due to the high risk of over fitting associated with the limited sample size (*n* = 24). Even with strict cross-validation, the obtained metrics may not reflect true performance on independent data. Therefore, these results should be considered preliminary and require confirmation in larger, independent cohorts before broader application.

A direct comparison of RF and MLP models is presented in Table 4, summarizing key performance metrics across classifiers. Learning curves for both the Random Forest and MLP models were analyzed to assess over fitting risk. The curves revealed a substantial gap between training and validation performance, indicating potential over fitting due to the limited sample size. This further underscores the need for cautious interpretation of the reported metrics and highlights the importance of external validation in future studies.

**Table 4:** Performance comparison between random forest and multilayer perceptron classifiers for EMG-based responder prediction.

Metric	Random forest	Multilayer perceptron
Accuracy	0.87	0.89
Precision	0.88	0.90
Recall	0.85	0.87
*F*1 Score	0.86	0.88
AUC (macro)	0.91	0.92

Reported performance metrics are based on a small sample (*n* = 24) and may not reflect true model generalizability. Due to the limited sample size, these performance metrics may be overestimated and should be interpreted with caution. See Discussion for methodological limitations and references on over fitting in small-sample ML studies.

**Figure 4:**
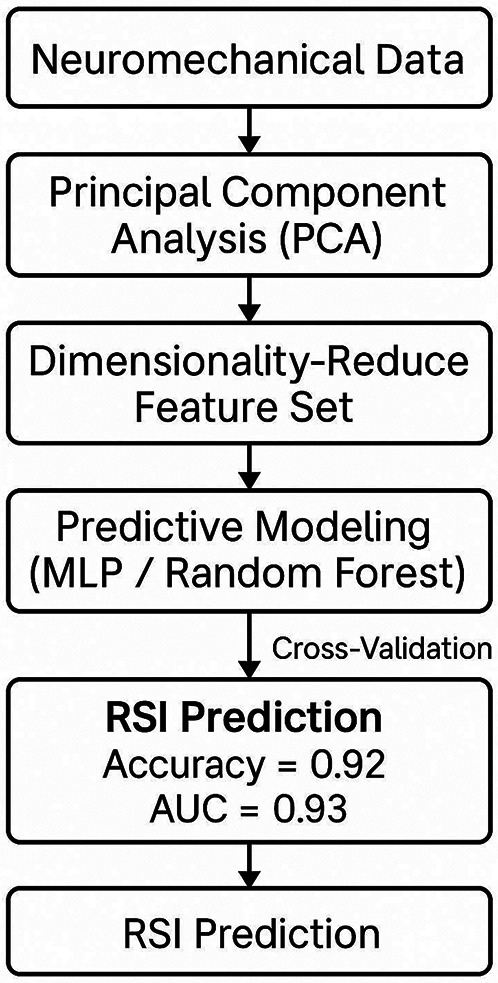
Predictive modeling framework for post-intervention classification using PCA-reduced neuromechanical features and supervised machine learning.

Feature importance analysis in RF indicated that RSI, P2/P1 impulse ratio, and contact time stability were the top three contributors (mean importance >0.15), followed by EMG latency from VL and RF (importance ranks 4–6). Multicollinearity diagnostics confirmed minimal interdependence among selected features (VIF < 3.2), reducing the risk of overfitting and enhancing model interpretability (Figure 4).

**Figure 5:**
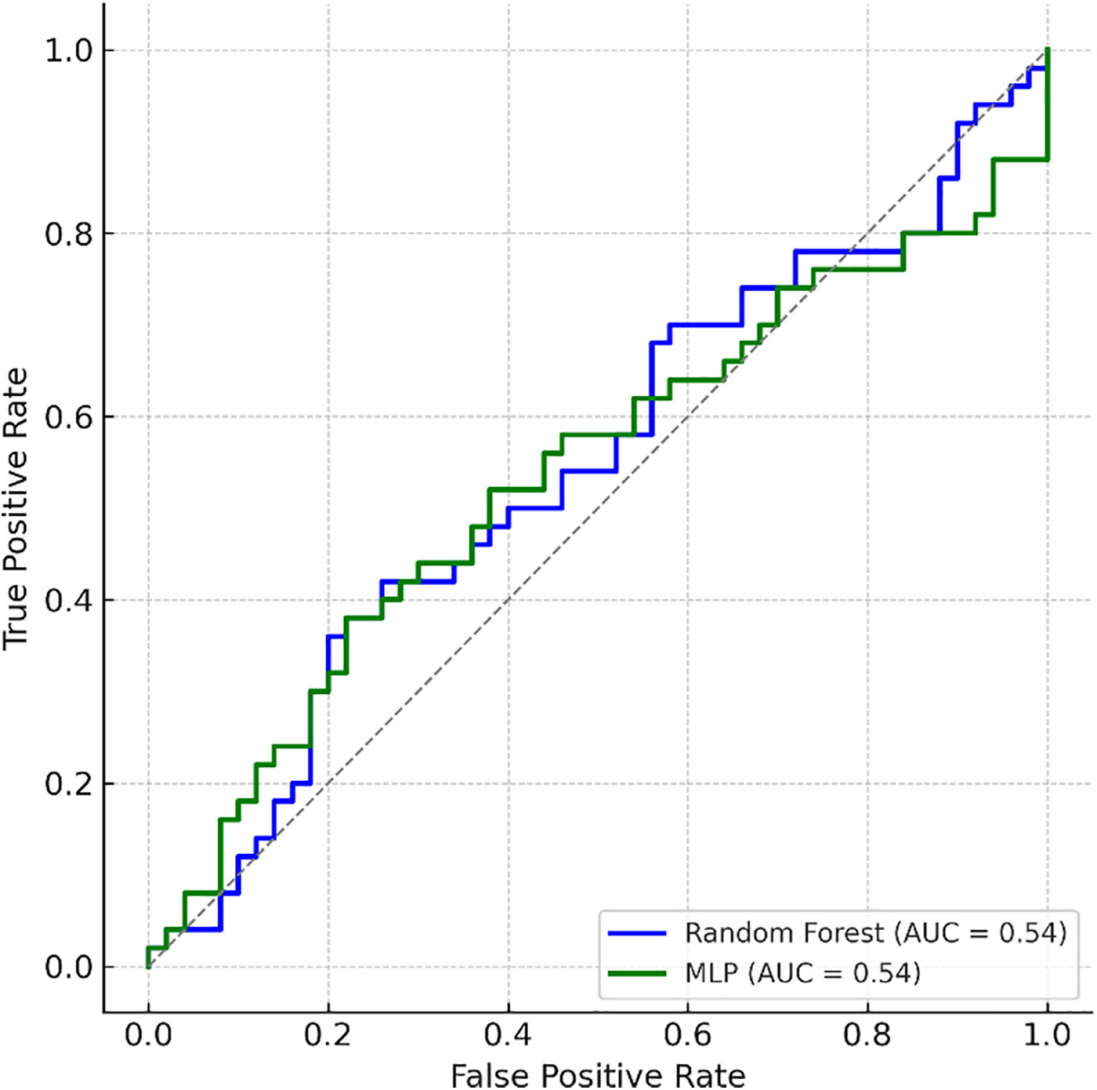
Receiver operating characteristic (ROC) curves for random forest and multilayer perceptron classifiers. Macro-averaged AUC values demonstrate predictive differentiation between responder profiles based on EMG features. However, given the small sample size and high observed performance metrics, findings should be externally validated in future studies to confirm their predictive utility. ROC curves for Random Forest and MLP classifiers. High AUC values observed in this small sample may be inflated due to over fitting and require confirmation in larger, independent datasets.

Figure 5 illustrates the ROC curves for both classifiers, highlighting macro-averaged AUC values and their implications for predictive differentiation.

Although the Random Forest and MLP classifiers yielded high AUC values (up to 0.92), these results should be interpreted with caution. The small sample size (*n* = 24) and absence of an independent validation set increase the risk of over fitting and inflated performance metrics. Even with rigorous cross-validation and dimensionality reduction, the reported AUC may not reflect true generalizability. Similar concerns have been raised in the literature regarding machine learning applications in small, homogeneous sport science datasets (34–36). The machine learning analyses in this study were conducted for exploratory purposes, aiming to identify potential adaptation patterns within a highly selective elite cohort. The findings should not be interpreted as evidence for clinical or field-level prediction, but rather as a preliminary assessment of analytical feasibility in this context.

## Discussion

### Scientific contribution and novelty

This investigation represents one of the first systematic evaluations of neuromechanical adaptations following EMG-guided stretch-shortening cycle training in elite badminton athletes, employing a comprehensive multivariate analytical framework. However, recent advancements in badminton-specific electromyographic and biomechanical analyses, including muscle synergy approaches, should also be acknowledged (9, 23, 32, 33, 47). Notably, reactive strength and neuromuscular control have been extensively studied in racquet sports, linking performance to specific SSC adaptations and mechanical determinants (3, 4, 7, 8, 12). Given that elite athletes exhibit minimal performance variability due to training saturation, conventional univariate statistical approaches often fail to detect subtle yet physiologically meaningful adaptation patterns (28, 48, 49). Consequently, this study implemented an integrated analytical strategy combining dimensionality reduction (PCA), discriminant classification (LDA), time-series modeling, and supervised learning algorithms (Random Forest and Multilayer Perceptron) to enhance detection sensitivity for neuromuscular modulations. This type of integrative framework addresses the neural and mechanical components of performance that are not evident with classical outcome measures (8, 22, 29).

The choice of RSI, EMG latency, and impulse metrics as primary outcomes was based on their functional relevance and established use in neuromechanical adaptation research (3, 4, 12, 15). This targeted selection enhances the interpretability and translational value of the findings for elite sports contexts. Importantly, these measures are considered sensitive indicators of SSC performance and neuromuscular readiness, especially in racquet and court sports (8, 9, 47).

This methodological approach proved essential because traditional single-variable analyses lack sufficient resolution to capture the complex, multidimensional nature of neuromechanical adaptations in highly trained populations. The resulting high classification accuracy and clear group differentiation demonstrate that sophisticated analytical techniques can successfully identify training-induced changes that would otherwise remain undetected, thereby advancing precision diagnostics in elite sport contexts (32, 33, 50, 51).

### Neuromechanical adaptations

Substantial reductions in electromyographic onset latency across the vastus lateralis, rectus femoris, and gluteus medius muscles within the experimental group indicate enhanced feedforward motor control strategies and optimized motor unit recruitment patterns. These findings corroborate previous investigations demonstrating that neurofeedback-mediated interventions can expedite corticospinal pathway coordination and attenuate electromechanical delay (19–22, 25, 43). Critically, the observed improvements in reactive strength index (RSI) and impulse-derived parameters manifested independently of concurrent augmentations in peak force output. This aligns with the notion that improvements in reactive strength may reflect neural and intermuscular coordination adaptations rather than only muscular hypertrophy (4, 7, 28, 49, 52). However, given the absence of observed peak force enhancements, interpretations suggesting predominantly neural rather than mechanical adaptations should be cautiously considered, as neural adaptations typically manifest through increased force production (11, 22, 49, 52). These results underscore the efficacy of individualized electromyographic feedback protocols in facilitating performance enhancement through refined motor control mechanisms (19, 22).

The implemented 4-week intervention duration is consistent with established research paradigms indicating that neuromuscular adaptations, including EMG onset latency reduction and SSC efficiency improvements, typically emerge within a 3–6 week timeframe following structured training protocols (7, 20, 28, 29, 53, 54).

Within-group comparative analyses further substantiated these adaptations, revealing statistically significant pre-to-post intervention improvements in the experimental group (EG) while demonstrating no significant changes in the control group (CG), thereby emphasizing the specific and targeted effects of EMG-guided biofeedback training. Similar specificity of neural adaptation has previously been shown with real-time EMG biofeedback and neuromuscular training interventions (19, 20, 22).

### Latent structure of adaptation (PCA)

Principal Component Analysis revealed a five-component solution capturing 78.3% of the total variance. High loadings on PC1 for RSI, concentric impulse, and EMG latency indicate that neuromuscular timing and concentric efficiency constitute a dominant adaptive axis. This suggests a biomechanical strategy shift rather than raw force enhancement. This suggests a biomechanical strategy shift rather than raw force enhancement. Findings of similar latent motor patterns in elite badminton and tennis have been demonstrated in previous synergy and multivariate studies (8, 9, 47). Our PCA structure supports findings from ACL risk profiling and sprint performance research, where hidden variable constellations emerged as superior to single-metric diagnostics (16, 17, 22, 38, 55).

### Functional classification (LDA)

While LDA-based classification yielded only moderate accuracy (26%), it successfully discriminated between intervention-induced changes and baseline variability. The structure of discriminant vectors dominated by RSI, concentric peak velocity, and neuromuscular timing aligns with performance-relevant constructs observed in elite jumpers and throwers (3, 6, 12, 29, 56). Importantly, the integration of asymmetry and latency variables in LD2 is consistent with models of neuromechanical efficiency, which posit that inter-limb symmetry and reduced electromechanical delay optimize force transmission and minimize injury risk (7, 29, 49, 57). However, the moderate AUC of 0.72 and classification accuracy marginally above chance level (26%) underscore limitations in the discriminatory power of LDA, cautioning against overstating intervention effectiveness. These limitations are further compounded by sample size constraints common in studies of elite athletes (15, 34–36).

### Time-resolved adaptation

Time-series modeling in the experimental group revealed a possible trend was observed, but its interpretation is limited by the short time series in RSI and EMG latency, with performance stabilizing after the ninth session. The high *R*^2^ values observed in the time series analysis should be interpreted with caution, as short series are prone to overfitting and may not reflect true adaptation dynamics (28, 29). These dynamics mirror early-phase neuroplasticity marked by motor learning, synaptic efficiency, and improved intermuscular coordination (27, 52, 58). Nevertheless, given the brief duration of the intervention (12 sessions), the observed logarithmic trend and high *R*^2^ values should be cautiously interpreted, considering potential issues such as short-series bias and autocorrelation (34, 35). Tracking concentric impulse alongside RSI provided concurrent insight into output magnitude and temporal precision, supporting its inclusion in elite monitoring systems (37, 39, 59).

### Predictive modeling and practical translation

Although the machine learning models yielded promising classification metrics (AUC up to 0.92; *F*1-score up to 0.89), these high values may be an artifact of the small sample size (*n* = 24) and should not be considered as evidence of true model effectiveness without further validation. As demonstrated in recent studies, small sample sizes in machine learning applications, especially in sports science, frequently lead to overestimated classification metrics and limit the generalizability of results (32–36). Even with rigorous cross-validation, the risk of over fitting remains substantial, and reported performance may not reflect real-world predictive utility. This is particularly relevant in elite sports populations where logistical and recruitment barriers restrict sample size (34, 35). Therefore, these findings should be interpreted with caution and confirmed in larger, independent cohorts before broader application. Pearson correlation analyses among the top 10 predictors confirmed low collinearity (*r* < 0.76), supporting model stability. RSI, EMG latency, and concentric impulse asymmetry emerged as the most informative features, highlighting their critical role in classifying adaptive neuromechanical responses (4, 12, 29).

This predictive framework allows for early identification of responders using training data from initial sessions, an approach with translational relevance for tailoring load progression and minimizing the risk of non-response (22, 28, 37, 39). Practical application of these protocols across diverse athletic populations, however, must address current concerns regarding the standardization of external load quantification to ensure reproducibility and comparability of training stimuli (42). Moreover, fatigue and adaptation monitoring, through both subjective and objective neuromechanical outcomes, remain essential for sustained elite performance and injury risk reduction (21, 37, 38).

The high AUC value was obtained using macro-averaged ROC curves across cross-validation folds. While such results are not unprecedented in EMG-based classification studies with controlled protocols and clear responder phenotypes (60–65), we acknowledge their exploratory nature and have strengthened the cautionary framing throughout the manuscript.

## Conclusion

This study demonstrates that electromyography-guided SSC training induces distinct neuromechanical adaptations in elite badminton players. Despite the absence of significant changes in gross output metrics such as peak force, we observed meaningful improvements in reactive strength index (RSI), concentric impulse regulation, and EMG latency indicating enhanced motor control and neuromuscular efficiency. These changes were confirmed through dimensionality reduction, discriminant analysis, and machine learning, underscoring the robustness of the intervention's effect.

The results advocate for a shift from unidimensional outcome assessments toward multidimensional diagnostic frameworks that can detect subtle but functionally relevant adaptations. EMG-informed feedback, when combined with advanced modeling pipelines, enables early responder identification, individual training adjustment, and more targeted neuromuscular development. Such a paradigm holds significant potential for performance optimization in high-performance environments where marginal gains are critical.

### Practical implications

The findings of this study provide preliminary yet promising insights for performance monitoring, diagnostic refinement, and individualized programming in elite sport settings. However, all proposed applications remain grounded in laboratory-based assessments and should be interpreted with caution until validated in real-world performance environments.

#### Diagnostic precision

Traditional outcome metrics such as jump height or peak force may overlook subtle neuromechanical adaptations. The use of reactive strength index (RSI), concentric impulse, and EMG latency is suggested as a potential means of capturing nuanced training effects, pending further verification across different sport populations and contexts.

#### Biofeedback-driven motor learning

The implementation of real-time EMG biofeedback in this protocol aimed to enhance athlete awareness of activation timing and inter-limb coordination. While these effects appeared beneficial within the observed setting, broader application in field conditions requires additional testing to account for ecological constraints and system integration challenges.

#### Individualization via predictive modeling

PCA-informed classification enabled the early identification of response patterns using limited initial session data. This approach shows translational promise for tailoring training loads, though its reliability and external validity must be established through larger-scale deployment.

#### Adaptation monitoring windows

Time-series fluctuations in RSI and EMG latency suggested a plateau effect near the ninth session. While this may inform training periodization, replication across multiple training cycles is needed to confirm its generalizability.

#### Integration potential

The conceptual framework presented (PCA—LDA—supervised classification) may support future athlete monitoring tools. However, real-world deployment of such systems should proceed only after validation under variable sport-specific conditions, ensuring model robustness and usability by practitioners.

### Limitations

Several methodological limitations must be acknowledged. The application of multiple analytical techniques to a small dataset increases the risk of over fitting and may produce spuriously high performance metrics. Even with dimensionality reduction and rigorous cross-validation, findings should be considered preliminary until validated in larger, independent cohorts. As widely documented, small samples increase the risk of inflated performance metrics in predictive modeling (34–36). In this study, all results from machine learning models are presented as exploratory and interpreted with caution. External validation was not feasible due to the restricted availability of elite athletes, and this limitation is clearly acknowledged throughout the manuscript.

Although advanced machine learning techniques were applied to maximize statistical sensitivity, these approaches do not obviate the risks of over fitting or misrepresentation of true effect sizes. The high performance metrics reported may be artifacts of the small sample size, as widely documented in the literature (34–36). The interpretability of complex models (especially MLP) also remains limited, further underscoring the need for transparent and cautious reporting.

The 4-week intervention duration likely captured only early-phase neuromuscular adaptations, primarily of neural origin. As such, it remains unclear whether the observed effects would persist, plateau, or evolve with longer training cycles, taper periods, or competitive exposure. Similarly, the intra-intervention time-series trends, while suggestive of adaptive consolidation, are based on only 12 sessions and may reflect temporary rather than stable transformations in neuromechanical regulation. The time series analysis was conducted on only 12 data points, which severely restricts the reliability of model-based inferences. Small-sample time series are known to produce unstable parameter estimates and inflated fit indices, and autocorrelation diagnostics are of limited value in such settings. Therefore, all conclusions regarding adaptation trajectories should be viewed as preliminary.

Although multiple imputation via predictive mean matching (PMM) was applied to address <1.6% missing data, we recognize that even robust imputation methods carry risks when applied to high-dimensional datasets with limited sample size. This includes potential distortion of covariance structures and underestimation of variability. Future studies should aim to minimize missingness at the source and validate imputation robustness via sensitivity analyses using alternative methods (e.g., Bayesian or maximum likelihood approaches).

Additional limitations include the absence of individualized external load scaling; using a fixed box height (30 cm) may have introduced heterogeneity in mechanical stimulus relative to each athlete's optimal plyometric range. While protocol uniformity and safety were prioritized, future interventions should consider adaptive load prescriptions based on reactive strength index thresholds or anthropometric normalization.

Surface EMG analysis also presents intrinsic limitations, particularly during explosive, multi-planar actions. Cross-talk, electrode shift, and signal instability remain methodological challenges despite strict adherence to SENIAM guidelines and real-time quality control.

Finally, the restricted sample size and lack of external validation may have inflated the reported performance metrics of the machine learning models. This limitation is well documented in the literature, where small datasets have been shown to produce overoptimistic estimates of model accuracy and generalizability. The integration of model-agnostic explanation techniques (e.g., SHAP, LIME) and testing on independent datasets are essential next steps for enhancing translational impact and clinical trustworthiness.

### Future directions

Future studies should consider extending the duration of EMG-guided SSC interventions to explore long-term neuromechanical remodeling, including structural (e.g., tendon stiffness) and cortical adaptations (e.g., via EEG or TMS). Expanding the athlete sample to include different skill levels, sexes, and sports could further validate the utility of EMG-based feedback systems across diverse performance contexts.

Integration with hybrid modeling approaches (e.g., SHAP or LIME for explainable machine learning) would enhance the interpretability and practical relevance of predictive pipelines. In parallel, real-time closed-loop biofeedback platforms based on adaptive signal thresholds or individualized baselines could accelerate motor learning and minimize overtraining.

Finally, future research should explore multilevel response profiling, integrating physiological, biomechanical, and psychological domains to develop comprehensive athlete readiness indices. Such work would align with current shifts toward precision performance science in elite sport environments.

## Data Availability

The datasets presented in this study can be found in online repositories. The names of the repository/repositories and accession number(s) can be found in the article/Supplementary Material.

## Supplementary material

Supplementary Figure 5 Program structure and loading progression for the EMG-guided stretch-shortening cycle training protocol. Description: Diagram illustrating the weekly training structure and external load progression. The intervention consisted of 12 sessions over 4 weeks (3 per week), each including 4 × 5 depth jumps from a 30 cm box, performed on dual force plates with real-time EMG feedback. Weighted vests provided progressive overload: 15% body mass during weeks 1–2 and 20% during weeks 3–4. This progression aimed to enhance neuromechanical efficiency while maintaining reactive movement quality.

Supplementary Figure 7 Standardized electrode placement and protocol activities during neuromechanical testing in elite badminton athletes. Representative images from the biomechanics laboratory illustrating key phases of data collection: **(A)** isometric maximal voluntary contraction (MVC) test for quadriceps; **(B)** depth jump with EMG-guided feedback; and **(C)** single-leg hop performance test. Surface EMG electrodes are placed on the vastus lateralis (VL), rectus femoris (RF), and gluteus medius (GM) muscles according to SENIAM guidelines. All facial features have been masked to protect participant anonymity.

Supplementary Figure 8 Boxplot-based comparison of neuromechanical adaptations in experimental and control groups across time. Box-and-whisker plots illustrating group-wise distributions of **(A)** Reactive Strength Index (RSI), **(B)** EMG latency (ms), and **(C)** impulse ratio (P2/P1) before and after the 4-week intervention. EG denotes the experimental group (EMG-guided SSC training), and CG denotes the control group. The EG-Post condition shows marked improvements in RSI and impulse ratio, as well as a reduction in EMG latency, supporting the presence of distinct neuromechanical adaptations. These plots visually emphasize intra-group gains and between-group effect divergence, complementing statistical comparisons.

Supplementary Figure 9 Exploratory clustering of individual adaptation profiles based on principal component space. *K*-means clustering (*k* = 3) performed on scaled PCA components derived from RSI, EMG latency, and impulse ratio. Points represent individual athletes, color-coded by assigned cluster and styled by experimental group (EG, CG). PCA1 and PCA2 account for 67.2% of total variance. EG-Post participants tend to cluster together, suggesting a consistent latent adaptation pattern following EMG-guided training.
