# Supplementary material for: Neuromechanical adaptations to EMG-guided SSC training in elite badminton players: a predictive multivariate approach
Source: Front Sports Act Living. 2025 Sep 11;7:1634656. doi: 10.3389/fspor.2025.1634656 (PMC12460308; doi:10.3389/fspor.2025.1634656)
Supplement: Supplementary file 1 [file Supplementaryfile1.zip › Supplementary Table S1&2.docx]

Supplementary Table S1: EMG Feature Testing

| Muscle | Latency Change (ms) | RMS Change (%) | Peak Amplitude Change (%) | p-value | Effect Size (d) |
| --- | --- | --- | --- | --- | --- |
| VL | -12.5 | 8.4 | 9.2 | 0.004 | 0.92 |
| RF | -10.3 | 7.9 | 8.8 | 0.006 | 0.87 |
| GM | -11.1 | 6.7 | 7.5 | 0.008 | 0.84 |

Supplementary Table S2. Summary of selected studies involving SSC-based or EMG-guided neuromuscular training in athletic populations.

| Study | Population | Duration | Key Measures | Main Outcomes | Comparison to Present Study |
| --- | --- | --- | --- | --- | --- |
| *García-Pinillos et al. (2018)* | Sub-elite sprinters (n = 21) | 6 weeks | CMJ, RSI, RFD | ↑RSI (+14%), ↑RFD | No EMG data; confirms SSC-based jump gains |
| *Wirth et al. (2016)* | Trained males (n = 28) | 8 weeks | EMG (VL, BF), squat 1RM | ↑EMG-RMS, ↑1RM | Similar neuromuscular trend, no predictive modeling |
| *Miyamoto et al. (2019)* | Youth athletes (n = 16) | 4 weeks | EMG latency (VL), sprint time | ↓latency (−12 ms), ↑speed | Focus on reactivity; aligns with latency reduction |
| *Baudry et al. (2021)* | Collegiate fencers (n = 18) | 5 weeks | SSC efficiency, pre-activation | ↑SSC-E (+17%), ↓co-contraction | Mechanistic insight, no ML framework |
| Present study | Elite junior badminton (n = 24) | 4 weeks | EMG (latency, RMS), SSC-E, PCA, LDA, MLP | ↓latency, ↑RMS, macro-AUC = 0.92 | Combines SSC and EMG with ML classification |
