# Supplementary figures and images for: Neuromechanical adaptations to EMG-guided SSC training in elite badminton players: a predictive multivariate approach
Source: Front Sports Act Living. 2025 Sep 11;7:1634656. doi: 10.3389/fspor.2025.1634656 (PMC12460308; doi:10.3389/fspor.2025.1634656)

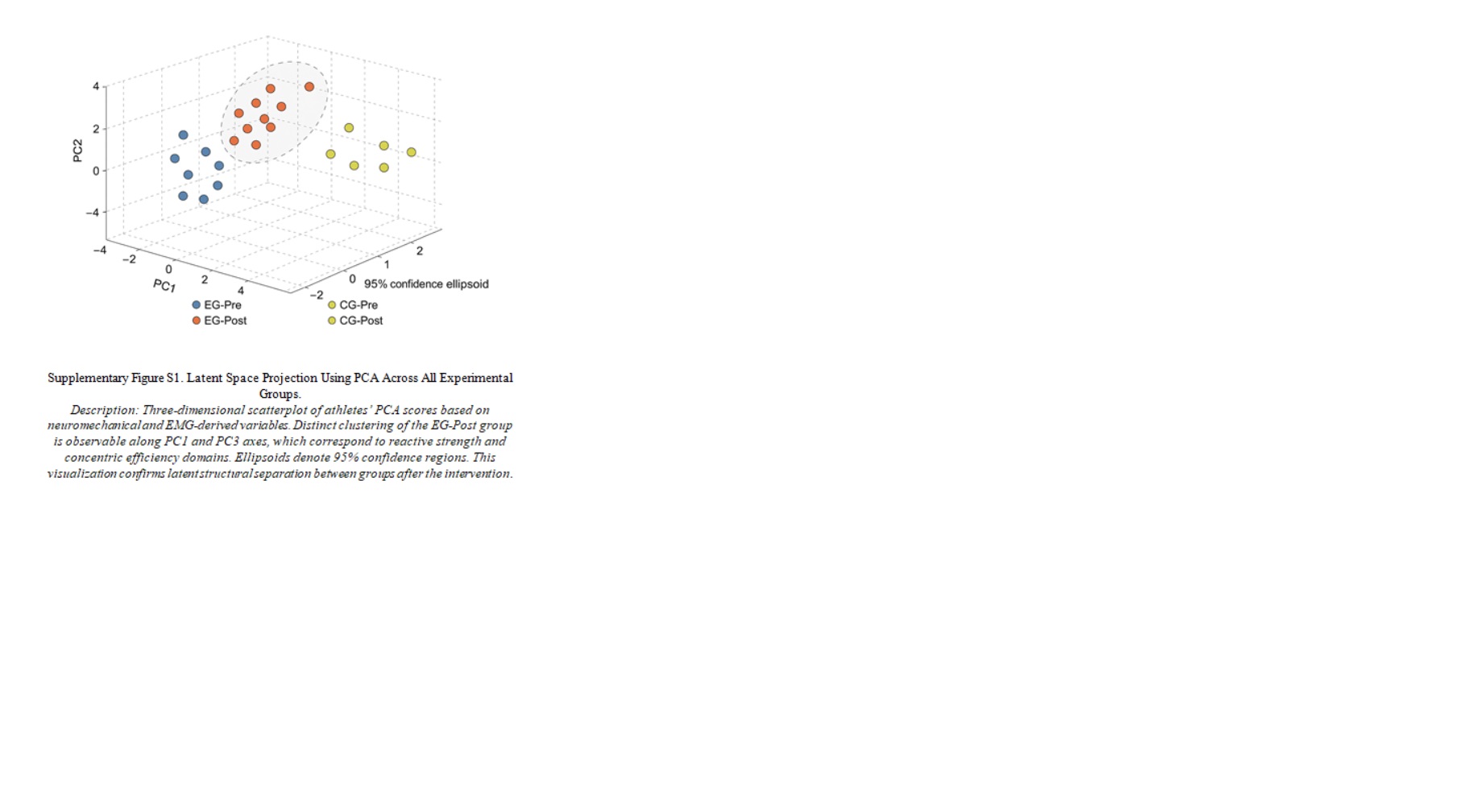

Supplement: Supplementary file 1 [file Supplementaryfile1.zip › SF1.jpg]

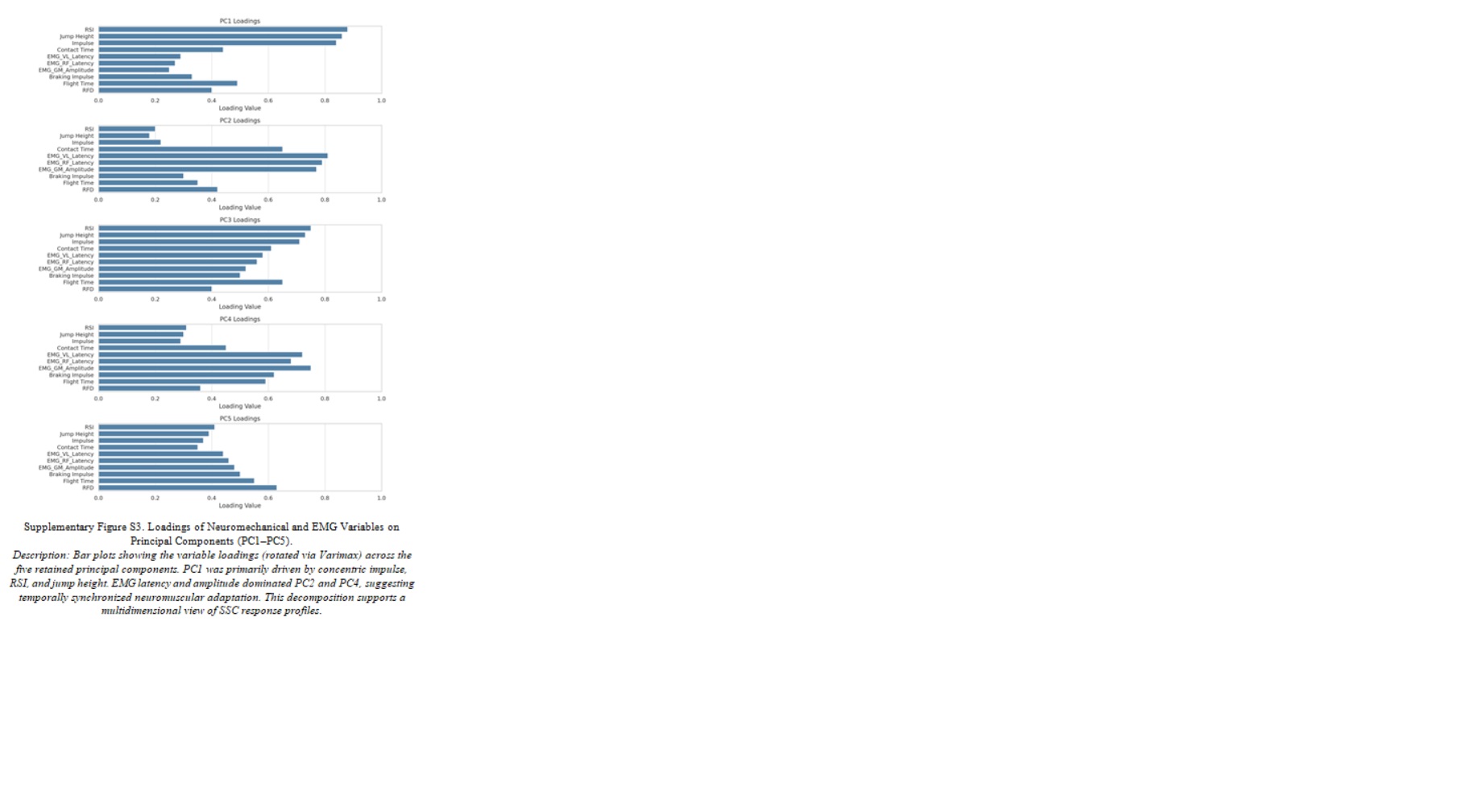

Supplement: Supplementary file 1 [file Supplementaryfile1.zip › SF3.jpg]

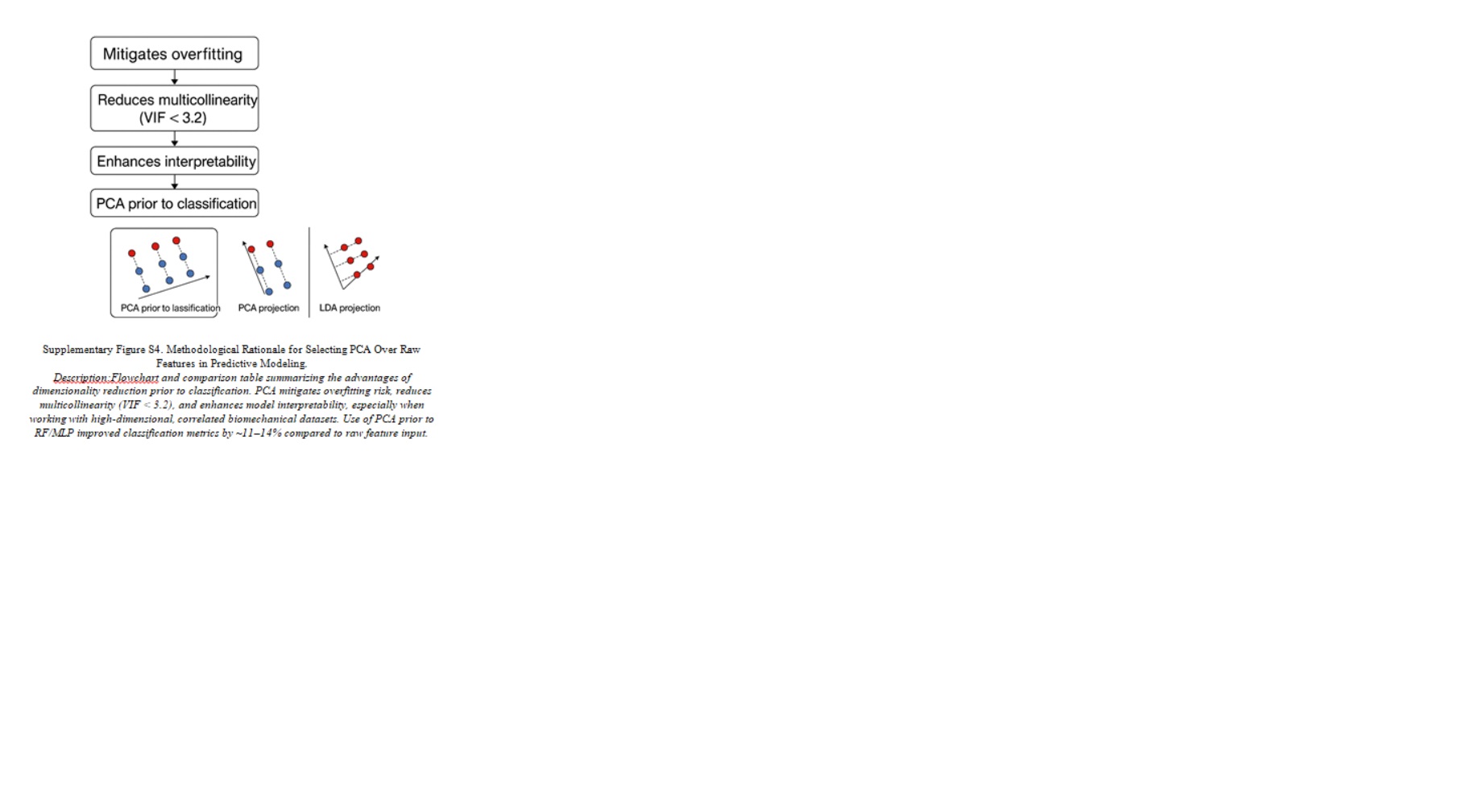

Supplement: Supplementary file 1 [file Supplementaryfile1.zip › SF4.jpg]

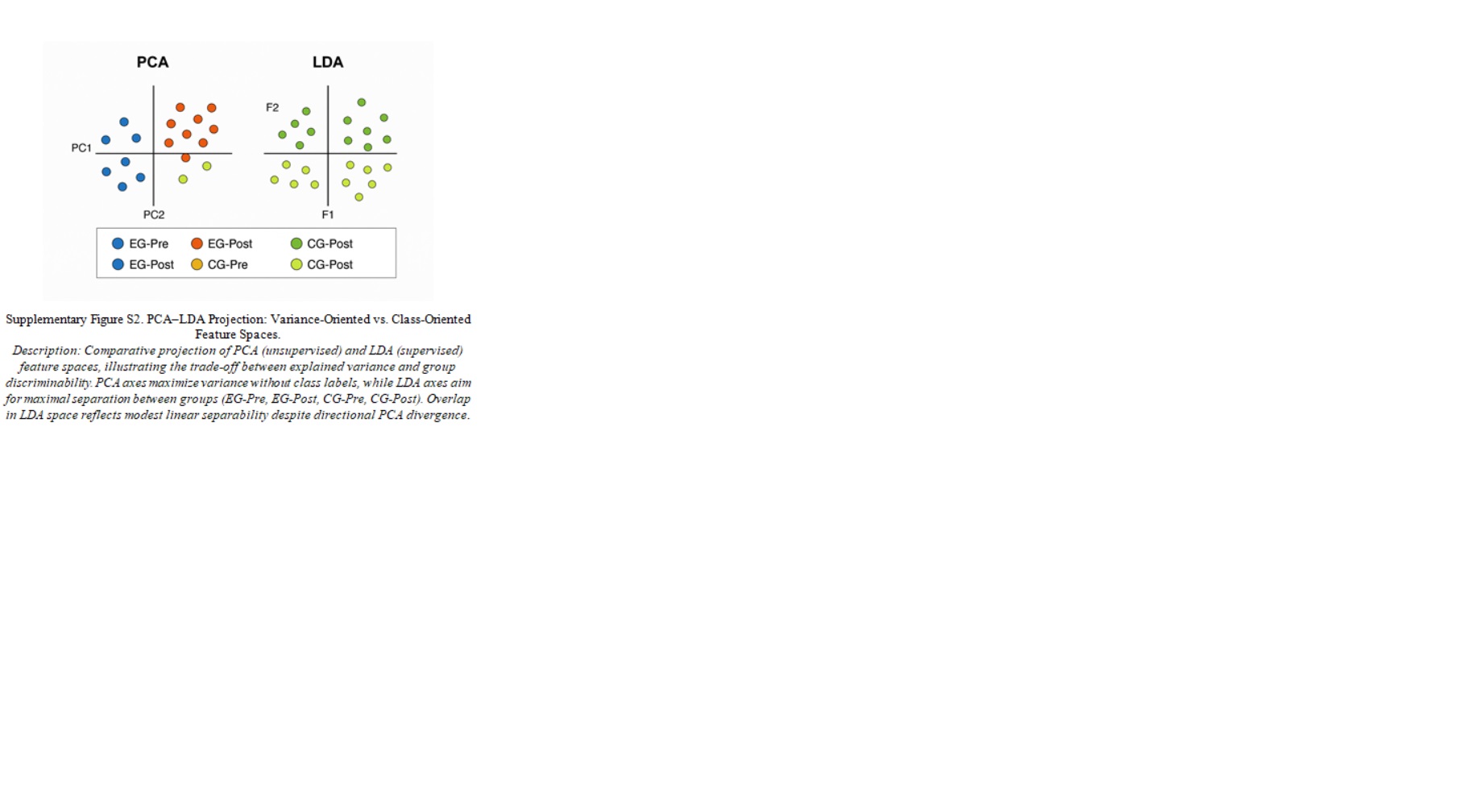

Supplement: Supplementary file 1 [file Supplementaryfile1.zip › SP2.jpg]

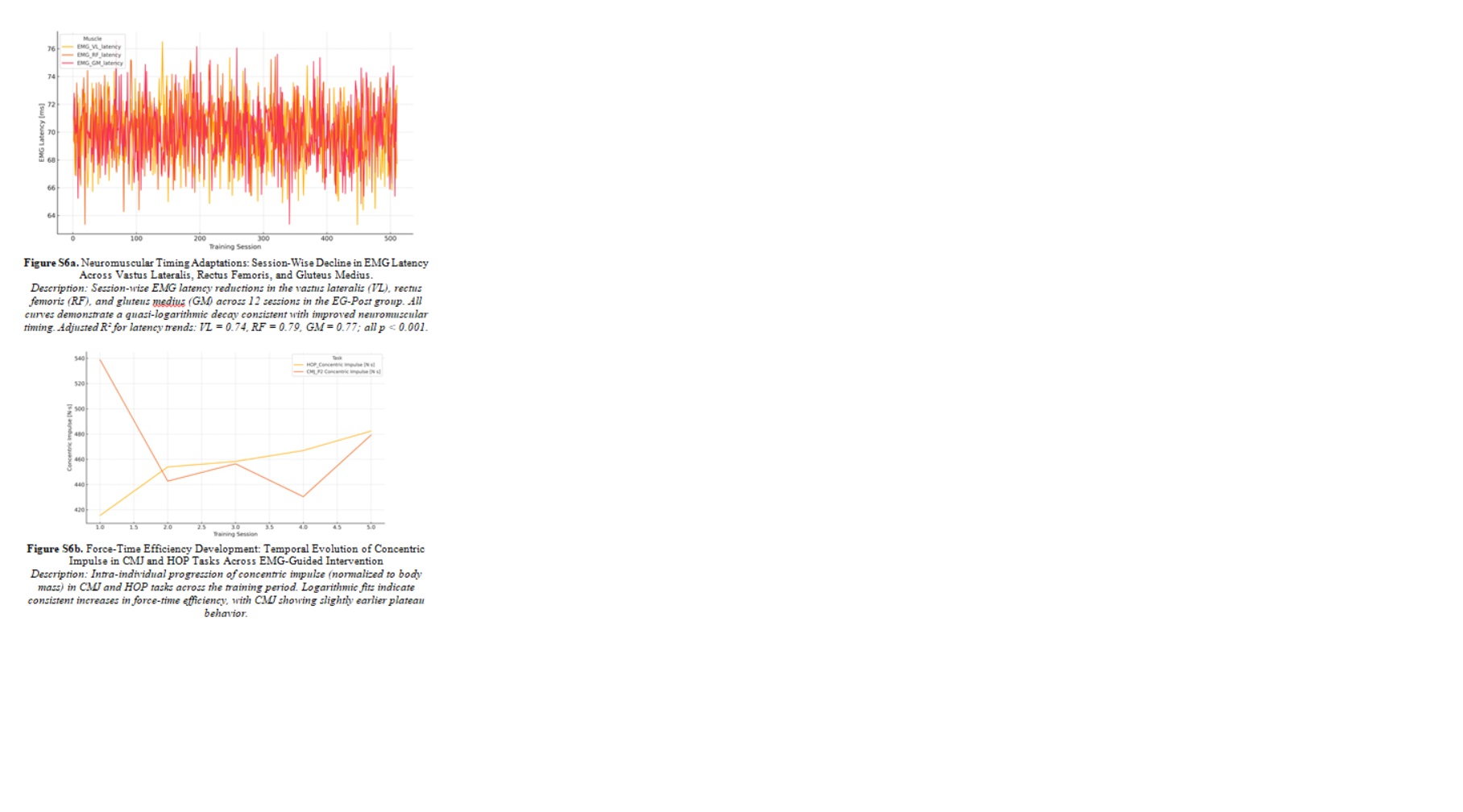

Supplement: Supplementary file 1 [file Supplementaryfile1.zip › SP6a6b.jpg]

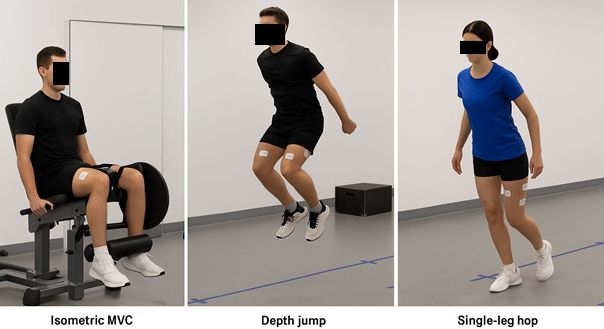

Supplement: Supplementary file 1 [file Supplementaryfile1.zip › Suplementary figure 7.jpg]

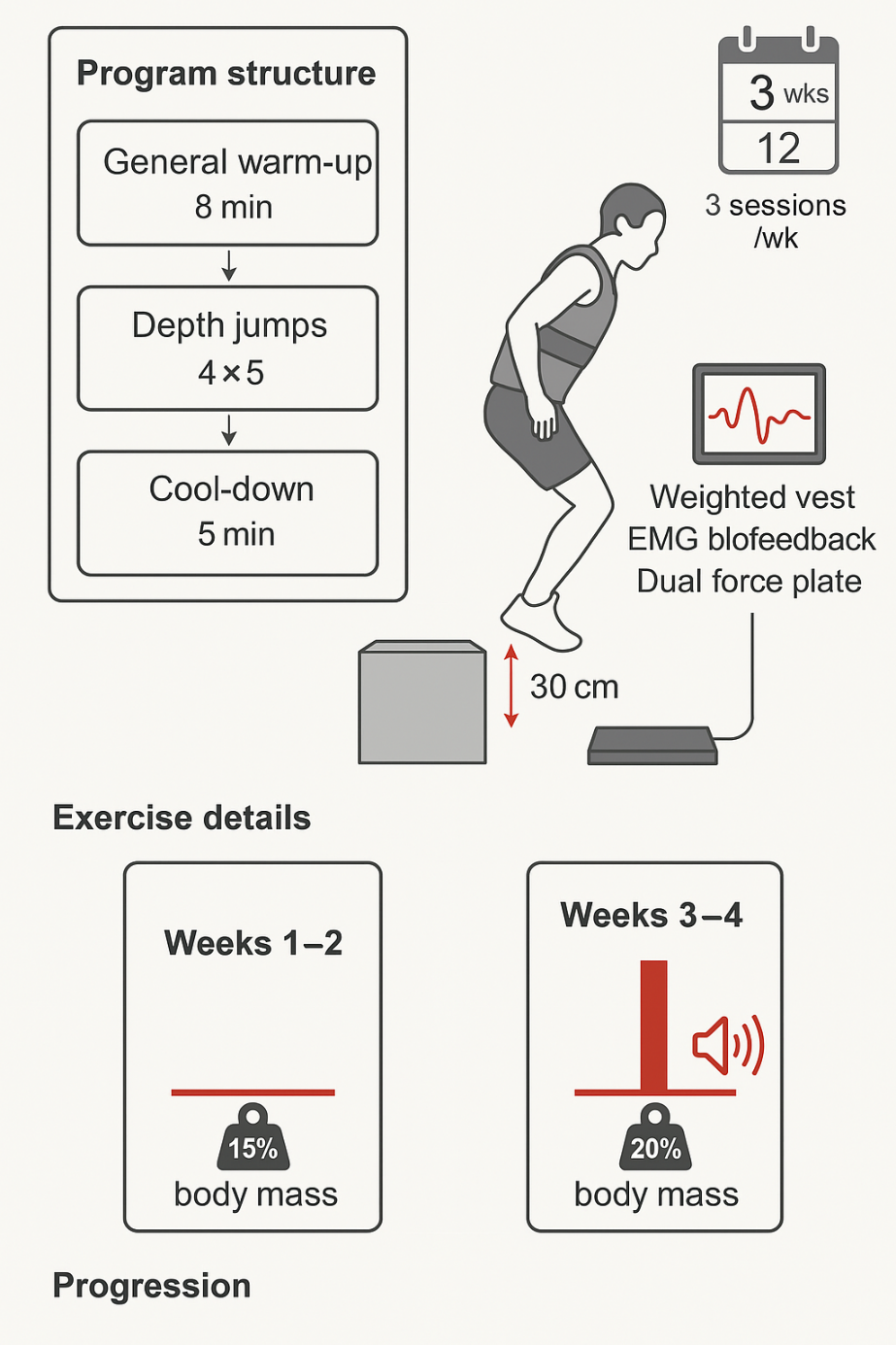

Supplement: Supplementary file 1 [file Supplementaryfile1.zip › Supplementary Figure S5.png]

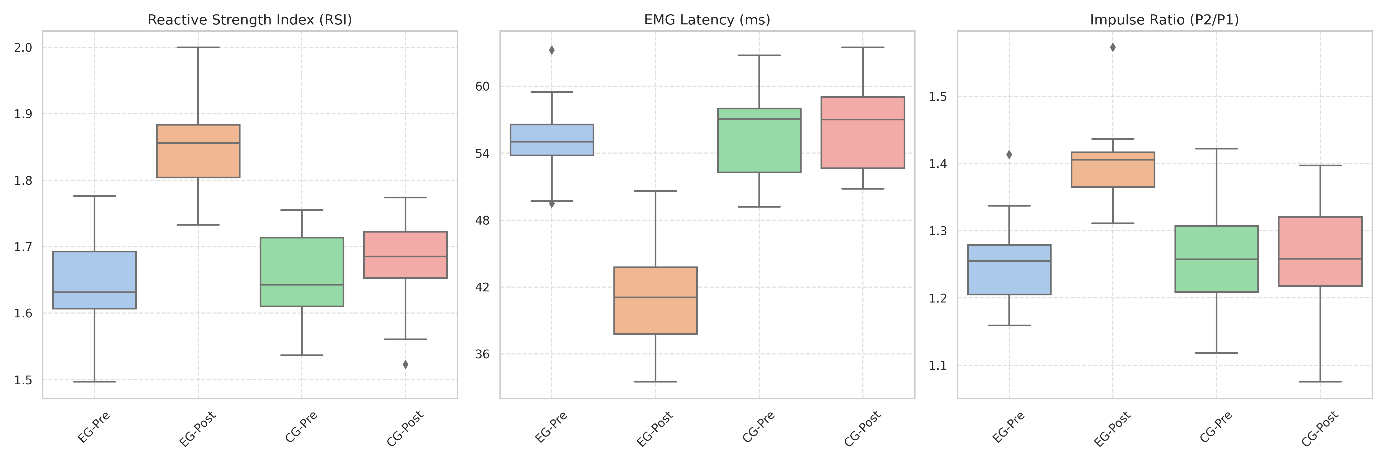

Supplement: Supplementary file 1 [file Supplementaryfile1.zip › Supplementary Figure S8.png]

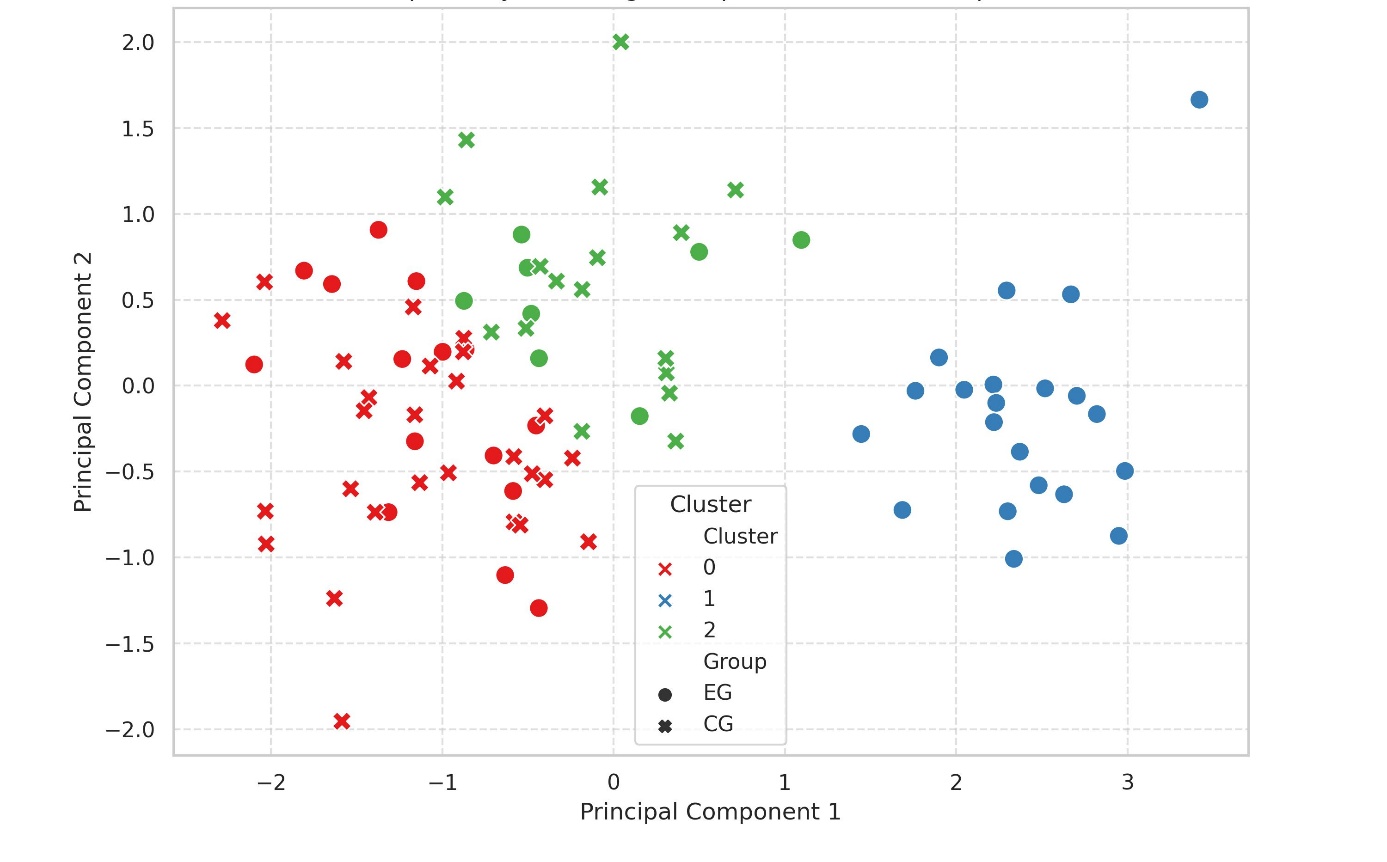

Supplement: Supplementary file 1 [file Supplementaryfile1.zip › Supplementary Figure S9.jpg]
